# Autophagy Plays an Essential Role in Mediating Regression of Hypertrophy during Unloading of the Heart

**DOI:** 10.1371/journal.pone.0051632

**Published:** 2013-01-07

**Authors:** Nirmala Hariharan, Yoshiyuki Ikeda, Chull Hong, Ralph R. Alcendor, Soichiro Usui, Shumin Gao, Yasuhiro Maejima, Junichi Sadoshima

**Affiliations:** Department of Cell Biology and Molecular Medicine, Cardiovascular Research Institute, University of Medicine and Dentistry of New Jersey, New Jersey Medical School, Newark, New Jersey, United States of America; Tokai University, Japan

## Abstract

Autophagy is a bulk degradation mechanism for cytosolic proteins and organelles. The heart undergoes hypertrophy in response to mechanical load but hypertrophy can regress upon unloading. We hypothesize that autophagy plays an important role in mediating regression of cardiac hypertrophy during unloading. Mice were subjected to transverse aortic constriction (TAC) for 1 week, after which the constriction was removed (DeTAC). Regression of cardiac hypertrophy was observed after DeTAC, as indicated by reduction of LVW/BW and cardiomyocyte cross-sectional area. Indicators of autophagy, including LC3-II expression, p62 degradation and GFP-LC3 dots/cell, were significantly increased after DeTAC, suggesting that autophagy is induced. Stimulation of autophagy during DeTAC was accompanied by upregulation of FoxO1. Upregulation of FoxO1 and autophagy was also observed *in vitro* when cultured cardiomyocytes were subjected to mechanical stretch followed by incubation without stretch (de-stretch). Transgenic mice with cardiac-specific overexpression of FoxO1 exhibited smaller hearts and upregulation of autophagy. Overexpression of FoxO1 in cultured cardiomyocytes significantly reduced cell size, an effect which was attenuated when autophagy was inhibited. To further examine the role of autophagy and FoxO1 in mediating the regression of cardiac hypertrophy, *beclin1+/−* mice and cultured cardiomyocytes transduced with adenoviruses harboring shRNA-*beclin1* or shRNA-FoxO1 were subjected to TAC/stretch followed by DeTAC/de-stretch. Regression of cardiac hypertrophy achieved after DeTAC/de-stretch was significantly attenuated when autophagy was suppressed through downregulation of *beclin1* or FoxO1. These results suggest that autophagy and FoxO1 play an essential role in mediating regression of cardiac hypertrophy during mechanical unloading.

## Introduction

The postnatal heart undergoes hypertrophy in response to mechanical overload, which can be induced by high blood pressure or a partial loss of myocardial tissue after myocardial infarction. Cardiac hypertrophy is characterized by the enlargement of individual cardiomyocytes, expression of fetal-type genes, and cytoskeletal reorganization [1]. Although cardiac hypertrophy is an important adaptation of the heart in response to increased wall stress, the continued presence of hypertrophy leads to myocardial cell death and cardiac dysfunction, and thus, hypertrophy is believed to be a significant risk factor for the development of heart failure [2]. Importantly, however, cardiac hypertrophy can be reversed when the increased wall stress is normalized, a process termed regression. Unloading of hemodynamic stress by a left ventricular assist device induces regression of cardiac hypertrophy and improvement of LV function in end-stage heart failure patients [3]. Regression of cardiac hypertrophy is accompanied by activation of unique sets of genes, including fetal-type genes and those involved in protein degradation [4], [5]. However, the signaling mechanism mediating regression of cardiac hypertrophy has been poorly understood.

Macroautophagy (hereafter autophagy) is a bulk degradation process for cytosolic proteins and organelles mediated through the formation of double membranous vesicles, termed autophagosomes, fusion of autophagosomes with lysosomes, and degradation by the lysosomal acid hydrolases and proteases [6]. Autophagy is an important mechanism of catabolism for maintaining cellular homeostasis during energy deprivation, while it also contributes to the quality control of proteins and organelles during stress.

Although cardiac hypertrophy is often accompanied by increases in protein synthesis, it is also accompanied by structural remodeling and dysfunction of intracellular organelles. One of the cellular mechanisms for adaptation against these during cardiac hypertrophy could be activation of autophagy. In fact, autophagy is activated during acute and chronic phases of cardiac hypertrophy [7], [8]. Although the functional significance of autophagy during cardiac hypertrophy is not fully understood, autophagy may promote clearance of damaged proteins and organelles. Considering that regression of cardiac hypertrophy is the reverse process of hypertrophy, regression of cardiac hypertrophy may also influence autophagy.

The forkhead box, class O (FoxO) family transcription factors are critical regulators of autophagy in cardiomyocytes [9], [10]. They are also involved in regulating muscle atrophy in skeletal muscle via the activation of protein degradation mechanisms including autophagy and the ubiquitin proteasome system [11], [12], [13]. While FoxOs also negatively regulate hypertrophy in the heart [14], [15], whether FoxOs are directly involved in regression of cardiac hypertrophy and, if so, the precise mechanism through which FoxOs mediate regression of cardiac hypertrophy are unknown.

In this study, we used transverse aortic constriction followed by de-constriction as a model to study the mechanism mediating regression of cardiac hypertrophy. The goals of this study were to 1) elucidate the role of autophagy during regression of cardiac hypertrophy, and 2) understand the role of FoxO1 in mediating regression of cardiac hypertrophy. We here report that autophagy plays an important role in mediating regression of cardiac hypertrophy in response to mechanical unloading and that FoxO1 plays an important role in mediating autophagy and regression of cardiac hypertrophy.

## Materials and Methods

### Antibodies

Antibodies used in the study include those against LC3 (MBL, #PD014), p62 (ARP, #03-GP62-C), FoxO1 (Epitomics, #1874-1 & Cell Signaling, #9454), Beclin1 (BD Biosciences, #612112), Cathepsin L (Sigma-Aldrich, #C2970), Sirt1 (Upstate, #07-131), P-AMPKα (Cell signaling, #2535), AMPK (Cell Signaling, #2532), Rab7 (Sigma-Aldrich, #R4779) and α-Tubulin (Sigma-Aldrich #T6199).

### Microsurgery for pressure overload & unloading

The method of inducing pressure overload by TAC has been described previously [16]. To induce unloading of myocytes, the suture in the aortic arch was removed 1 week after TAC (DeTAC) and the mice were observed for 7 days, after which they were subjected to echocardiography and hemodynamic analyses and sacrificed. All protocols concerning animal use were approved by the Institutional Animal Care and Use Committee at the University of Medicine and Dentistry of New Jersey.

### Transgenic mice

Transgenic mice with cardiac-specific overexpression of WT-FoxO1 (Tg-FoxO1) were generated on an FVB background using the murine α-myosin heavy chain promoter provided kindly by Dr. J Robbins (University of Cincinnati, Cincinnati). The plasmid harboring WT-FoxO1 was a kind gift from Dr. Domenico Accili (Columbia University, New York) [17]. Transgenic mice expressing GFP-LC3 (Tg-GFP-LC3) [18], [19], Beclin1 heterozygous knockout mice (*beclin1+/−*) [18] and FoxO1 cardiac-specific homozygous knockout mice (c-FoxO1−/−) [9] have been described previously. Double transgenic or bigenic Tg-FoxO1 and Tg-GFP-LC3 mice were generated by breeding, and only F1 generation mice were used for evaluation of GFP-LC3 puncta.

### Echocardiography analyses

The method used to analyze cardiac function using echocardiography in mice has been described previously [9], [20].

### Primary culture of neonatal rat ventricular myocytes

Primary cultures of left ventricular cardiomyocytes were prepared from 1-day-old Crl: (WI) BR-Wistar rats (Harlan Laboratories) as described previously [9], [18]. A fraction enriched for cardiomyocytes (>95%) was obtained by centrifugation through a discontinuous Percoll gradient [18].

### Adenoviruses

Adenoviruses harboring WT-FoxO1 (Ad-FoxO1-WT), short hairpin (sh-) FoxO1 (Ad-sh-FoxO1), sh-scramble (Ad-sh-Scr), control LacZ (Ad-LacZ) [9], and sh-Beclin1 (Ad-sh-Beclin1) [9], [18] have been described previously. Myocytes were transduced with 15 MOI of adenoviruses. Ad-LacZ and Ad-FoxO1-WT transductions were carried out for 24 hours, while Ad-sh-Scr, Ad-sh-FoxO1 and Ad-sh-Beclin1 were transduced for 96 hours.

### Evaluation of cell size

To evaluate cardiomyocyte cross-sectional area *in vivo*, tissue sections of mouse hearts were fixed in 10% neutral-buffered formalin, embedded in paraffin, sectioned at 5 µm thickness and stained with wheat germ agglutinin Texas red, as described previously [20]. The outlines of at least 200 circular to oval shaped myocytes with nearly circular capillary profiles were traced in 10 fields from 3 different mouse samples and the cross-sectional area was measured using Image-Pro Plus software (Media Cybernetics). To determine cell size *in vitro*, images of cultured cardiomyocytes were obtained at 20× magnification using a light microscope. The outlines of cardiomyocytes obtained in 8 different visual fields from at least 3 different cultures were traced and the relative cross-sectional area was evaluated.

### Evaluation of fluorescent LC3 puncta

The method used to evaluate fluorescent LC3 puncta *in vivo* has been described previously [9], [18]. Briefly, fresh heart slices were embedded with Tissue-Tek OCT compound (Sakura Finetechnical Co., Ltd.) and frozen at −70°C. Sections 10-µm-thick were obtained from the frozen tissue samples using a cryostat (CM3050 S, Leica), air-dried for 30 min, fixed by washing in 95% ethanol for 10 minutes, mounted using a reagent containing 4′,6-diamidino-2-phenylindole (DAPI) (Vectashield; Vector Laboratories Inc.) and viewed under a fluorescence microscope (Nikon Eclipse E800). The number of GFP-LC3 dots was determined by manual counting in 10 fields from 5 different animals using a 60× objective, and nuclear number was evaluated by counting DAPI-stained nuclei in the same fields using the same magnification. The number of GFP-LC3 puncta/cell was evaluated as the total number of dots divided by the number of nuclei in each microscopic field.

### Sample preparation and immunoblot analysis

Heart tissue homogenates were prepared using RIPA buffer, while protein lysates from cultured cardiomyocytes were prepared using boiled 2× SDS sample buffer, as has been described previously [9]. To evaluate protein concentration in cultured myocytes, RIPA buffer with lysosomal protease inhibitors (1∶200 dilution) was used to extract proteins. The method used to detect protein expression using immunoblots has been described previously [9], [18]. Densitometric analyses of the blots were carried out using the public domain ImageJ program (NIH, Maryland).

### Quantitative reverse transcription-PCR

Total RNA was extracted from mouse hearts and *in vitro* cultures using TRIzol (Invitrogen). cDNA was synthesized using the RETROscript kit (Ambion) according to the manufacturer's instructions. Real time-PCR was carried out as stated previously [20]. Primers for Gabarapl1 [11], [13], Bnip3 [11], Ulk2 [13], and ANF [20] have been reported previously. The following primer pairs were also used –

FoxO1: Sense – CAGATCTACGAGTGGATGGT

FoxO1: Antisense – ACTTGCTGTGTAGGGACAGA

GAPDH: Sense – GAGCTGAACGGGAAGCTCACT

GAPDH: Antisense – TTGTCATACCAGGAAATGAGC

### *In vitro* model of hypertrophy regression

Neonatal rat ventricular myocytes were cultured in collagen-I-coated BioFlex plates (Flexcell Intl, #BF-3001C) and subjected to 20% cyclic stretch in a uniaxial strain at 30 cycles/min for 36 hours in a 37°C incubator. Myocytes subjected to mechanical stretch and incubation without stretch for 36 hours each were considered a de-stretch model. Control samples were cultured in the Bioflex plates without going through the stretch regimen.

### Autophagy Inhibitors

To inhibit autophagosome formation, cultured cardiomyocytes were treated with 10 mM 3-Methyladenine (3-MA) for 24 hours or Ad-sh-Beclin1 for 72 hours as described [18]. To inhibit autophagy flux *in vivo*, chloroquine was injected (10 mg/kg) intraperitoneally for 4 hours as previously described [21], following which animals were euthanized to detect expression of autophagy markers by immunoblot.

### Statistics

Data are expressed as mean ± SEM. Statistical analyses between groups of 2 were done by unpaired t-test. Groups of 3 or more were analyzed using one-way ANOVA, followed by the Newman-Keuls multiple comparison test. A value of p<0.05 was considered significant.

### Ethics Statement

All animal protocols were approved by the review board of the Institutional Animal Care and Use Committee of the University of Medicine and Dentistry of New Jersey (07115 and 10073).

## Results

### Left ventricular unloading induces regression of cardiac hypertrophy

**Figure 1:**
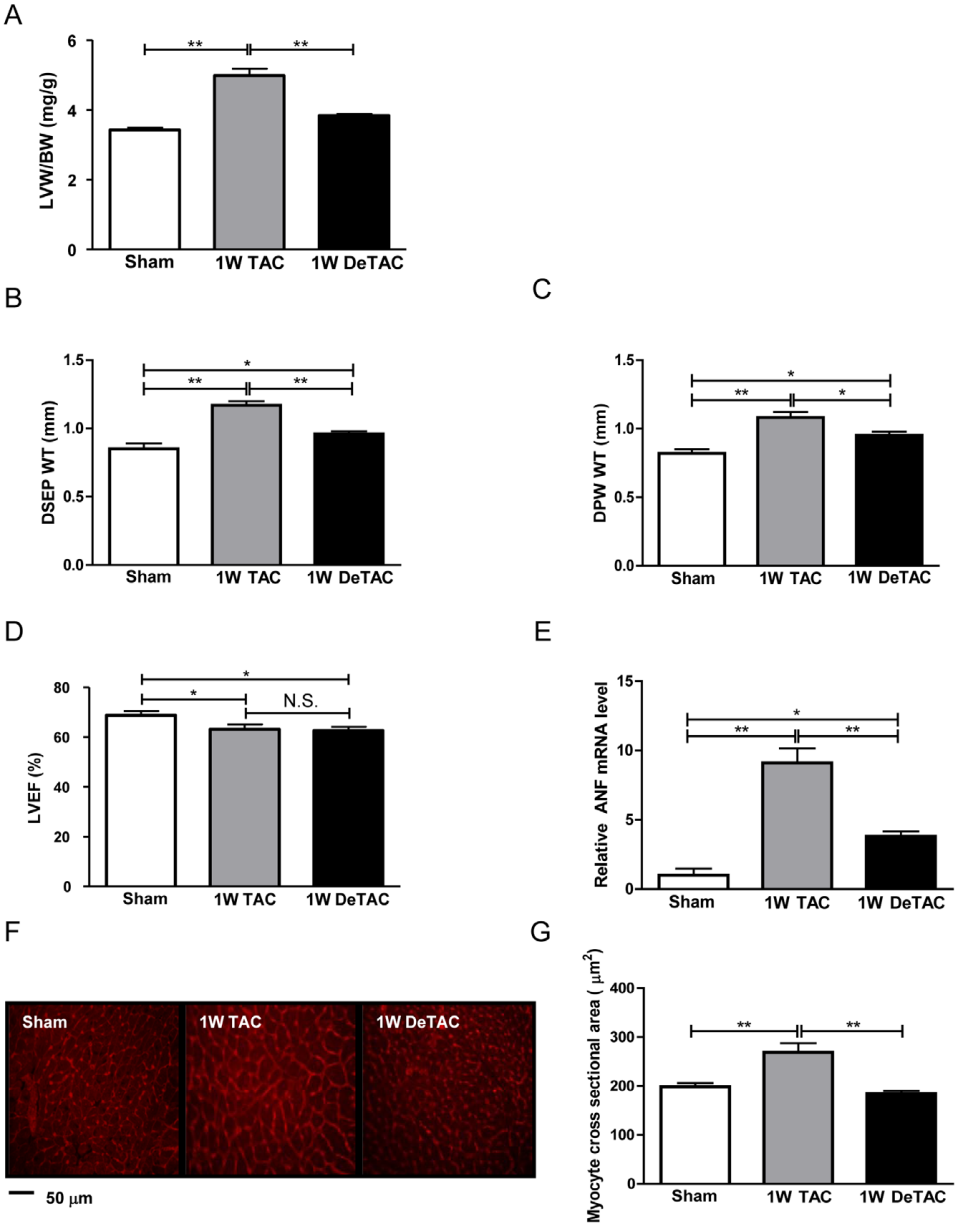
Left ventricular unloading induces regression of cardiac hypertrophy. C57BL/6 mice were subjected to pressure overload caused by thoracic aortic constriction for 1 week (1W TAC), followed by cardiac unloading by removal of the constriction for 1 week (1W DeTAC). A) Left ventricular weight/body weight (LVW/BW). B) Diastolic septal wall thickness (DSEP WT), C) diastolic posterior wall thickness (DPW WT), and D) left ventricular ejection fraction (LVEF), as evaluated by echocardiographic analyses. E) mRNA level of atrial natriuretic factor (ANF), evaluated by qRT-PCR. F) Representative images of transverse sections of the LV after wheat germ agglutinin staining. G) Cross-sectional area of myocytes (in µm^2^). N = at least 8 mice in each group. * p<0.05, ** p<0.01, N.S.: Not Significant.

To study the involvement of autophagy in the regression of cardiac hypertrophy, C57BL/6 mice were subjected to pressure overload caused by transverse aortic constriction for 1 week (1W TAC), after which unloading was induced by removal of the constriction for 1 week (1W DeTAC). After 1W TAC, cardiac hypertrophy was observed via echocardiographic and postmortem analyses, as indicated by significant increases in LV weight (LVW)/body weight (BW) (Fig. 1A), and diastolic septal and posterior wall thickness (Fig. 1BC). After 1W DeTAC, a significant decrease in cardiac mass was observed, indicating regression (Fig. 1ABC). Left ventricular ejection fraction (LVEF) was decreased slightly after 1W TAC compared to sham (Fig. 1D and Supplemental Table S1), but did not decrease further after 1W DeTAC (Fig. 1D). The mRNA level of atrial natriuretic factor (ANF), a fetal-type gene reactivated during cardiac hypertrophy, was increased significantly after 1W TAC and was decreased after 1W DeTAC (Fig. 1E). Histological analyses showed that LV myocyte cross-sectional area was increased after 1W TAC, whereas it was significantly and uniformly reduced after 1W DeTAC compared to after 1W TAC (Fig. 1FG and supplemental Fig. S1), again indicating regression of cardiac hypertrophy.

### Autophagy is enhanced during regression of cardiac hypertrophy

**Figure 2:**
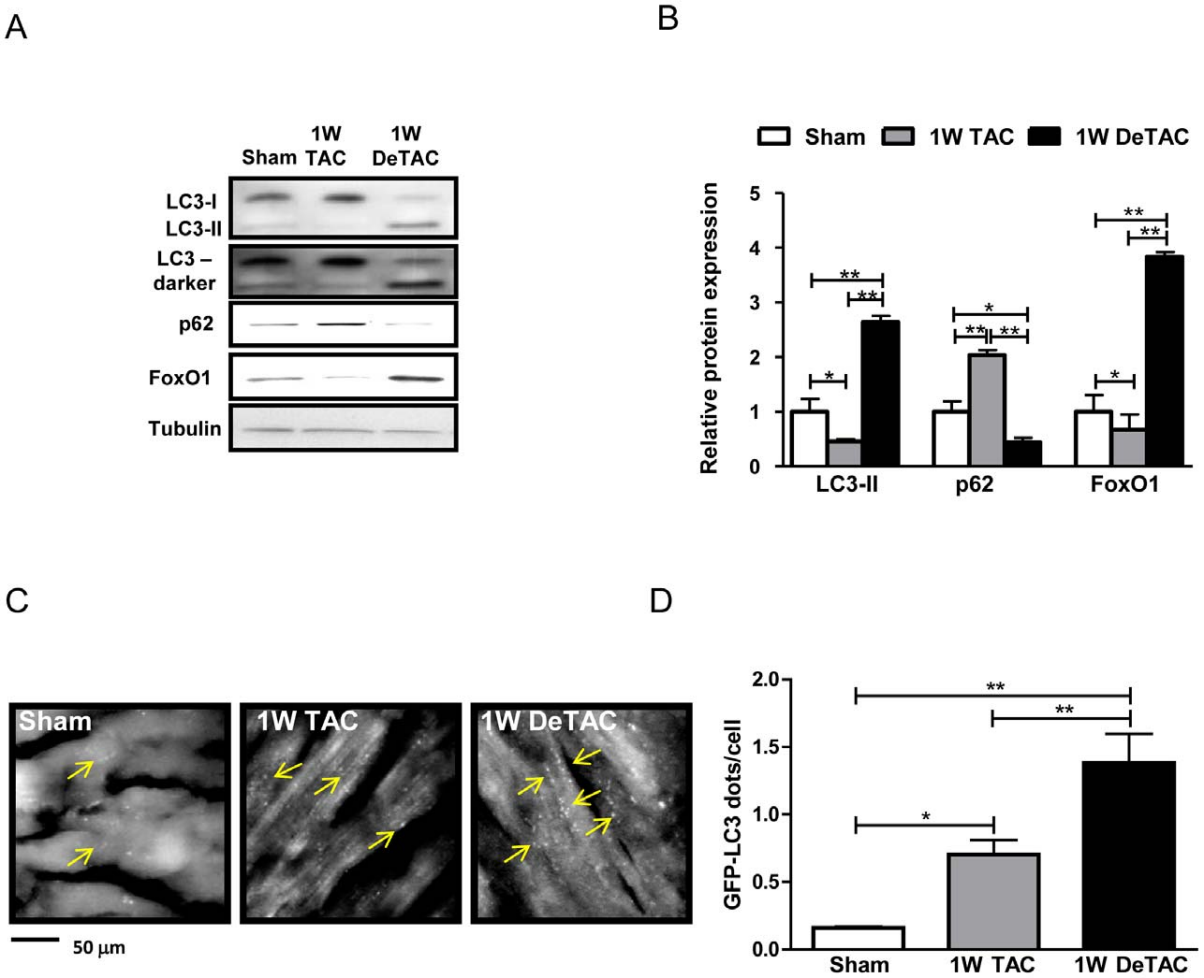
Autophagy and FoxO1 expression are upregulated during regression of cardiac hypertrophy. Control C57BL/6 and Tg-GFP-LC3 mice were subjected to sham, 1W TAC and 1W DeTAC surgeries. A) Representative immunoblots of autophagy markers and FoxO1 from mouse hearts. B) Densitometric analyses. C) Representative images of fluorescent LC3 puncta in hearts from Tg-GFP-LC3 mice. Arrows indicate LC3 puncta. D) Mean number of GFP-LC3 dots/cell. Data represent means from at least 3 mice each. * p<0.05, ** p<0.01.

We hypothesized that autophagy is activated during regression of cardiac hypertrophy. Expression of LC3-II, a protein known to be associated with autophagosomes, was increased following 1W DeTAC (Fig. 2AB). To evaluate the extent of autophagosome formation upon regression of cardiac hypertrophy, we examined transgenic mice harboring GFP-LC3 (Tg-GFP-LC3) [19] and found that the number of GFP-LC3 dots/cell was significantly increased upon cardiac unloading (Fig. 2CD). Expression of p62, a polyubiquitin-binding protein sequestered in autophagosomes for lysosomal degradation [22], was significantly reduced in DeTAC samples, indicating increased autophagic flux during regression of cardiac hypertrophy (Fig. 2AB). Collectively, these results suggest that autophagy is significantly induced during regression of cardiac hypertrophy. Interestingly, although the number of GFP-LC3 dots/cell was increased significantly, LC3-II was decreased and p62 was accumulated after 1W TAC. These results suggest that autophagic flux might be inhibited after 1W TAC under our experimental conditions (Fig. 2AB).

### FoxO1 is involved in mediating regression of cardiac hypertrophy

There are several reports describing the role of FoxO transcription factors in muscle atrophy [12], [13], [14], [15]. We and others have shown previously that FoxO1 is a critical regulator of autophagic flux in cardiomyocytes [9], [10]. Thus, we hypothesized that FoxO1 may be involved in mediating the reduction in cardiac mass and cell size during regression of cardiac hypertrophy. To this end, we evaluated expression of FoxO1 after 1W TAC and 1W DeTAC. FoxO1 was significantly upregulated during regression of cardiac hypertrophy, while it was downregulated during cardiac hypertrophy (Fig. 2AB).

### FoxO1 regulates autophagy *in vivo*

**Figure 3:**
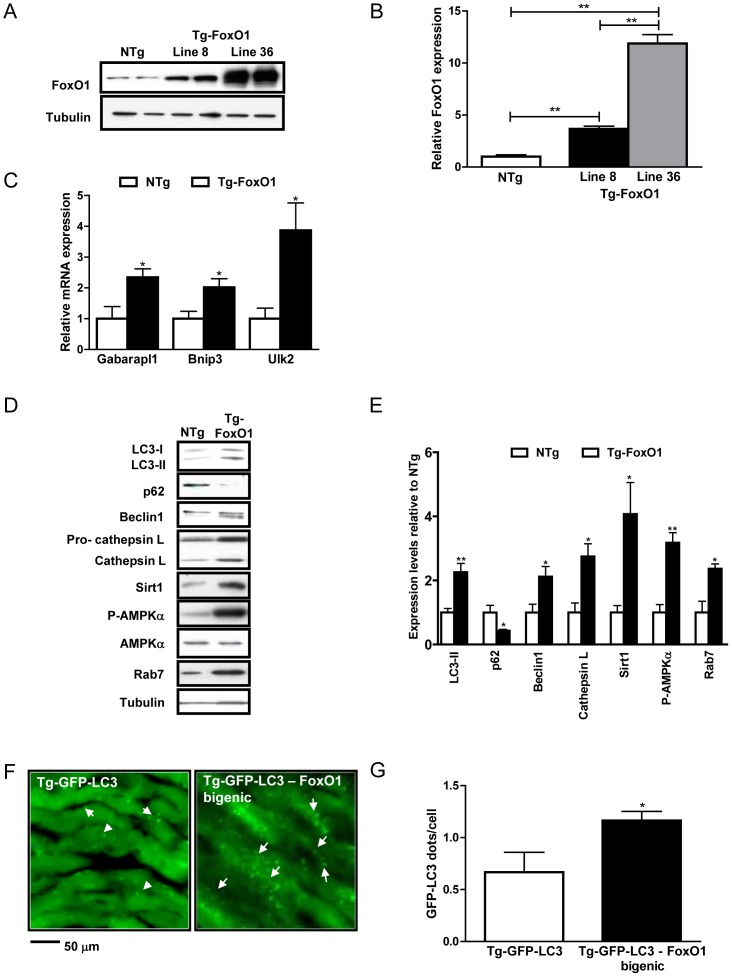
Overexpression of FoxO1 regulates autophagy *in vivo.* Transgenic mice with cardiac-specific overexpression of WT-FoxO1 (Tg-FoxO1) were generated. A) Representative immunoblots comparing FoxO1 expression levels in the two lines (lines #8 and #36) of Tg-FoxO1 and nontransgenic (NTg) mice. B) Densitometric analyses. C) mRNA levels of autophagy genes Gabarapl1, Bnip3 and Ulk2. D) Representative immunoblots of autophagy markers. E) Densitometric analyses. F–G) Tg-FoxO1 mice were bred with Tg-GFP-LC3 to generate Tg-GFP-LC3 – FoxO1 bigenic mice. F) Representative images of GFP-LC3 puncta. Arrows indicate LC3 puncta. G) Mean number of GFP-LC3 dots/cell. Data represent means from at least 4 individual mice. * p<0.05, ** p<0.01.

To elucidate the role of FoxO1 in mediating autophagy in the heart *in vivo*, we generated transgenic mice with cardiac-specific overexpression of WT-FoxO1 (Tg-FoxO1). We generated two lines of transgenic mice (lines #8 and #36) with different levels of FoxO1 expression (3.7- and 11.9-fold increase, respectively, relative to non-transgenic (NTg) mice) (Fig. 3AB). Both lines showed decreased cardiac mass, as evidenced by the significantly smaller size of the LV and the cardiomyocytes therein (Supplemental Fig. S2). In order to examine the effect of FoxO1 upregulation upon autophagy *in vivo*, we evaluated expression of several autophagy markers in Tg-FoxO1 mice (Line #8). mRNA levels of Gabarapl1, Bnip3, and Ulk2, genes associated with autophagosome formation [11], [13], were significantly increased in Tg-FoxO1 mice (Fig. 3C). Protein expression of autophagy markers and molecules known to regulate autophagy, including LC3-II accumulation, p62 degradation, Beclin1, Cathepsin L, Sirt1, P-AMPKα and Rab7, was significantly increased in Tg-FoxO1 mice (Fig. 3DE). To evaluate autophagosome formation, we generated bigenic mice (Tg-GFP-LC3-FoxO1) by breeding Tg-FoxO1 and Tg-GFP-LC3 mice. The number of GFP-LC3 dots/cell was significantly greater in the bigenic mice than in Tg-GFP-LC3 mice (Fig. 3FG), indicating that FoxO1 can increase autophagosome formation. Chloroquine treatment (10 mg/kg IP) further increased the level of LC3-II in Tg-FoxO1 as determined by immunoblot analyses (Supplemental Fig. S3). Together with the decreased expression of p62 (Fig. 3DE), these results suggest that FoxO1 positively regulates autophagy and autophagosome formation in the heart.

### Treatment with autophagy inhibitors attenuates FoxO1–induced reduction in cell size

**Figure 4:**
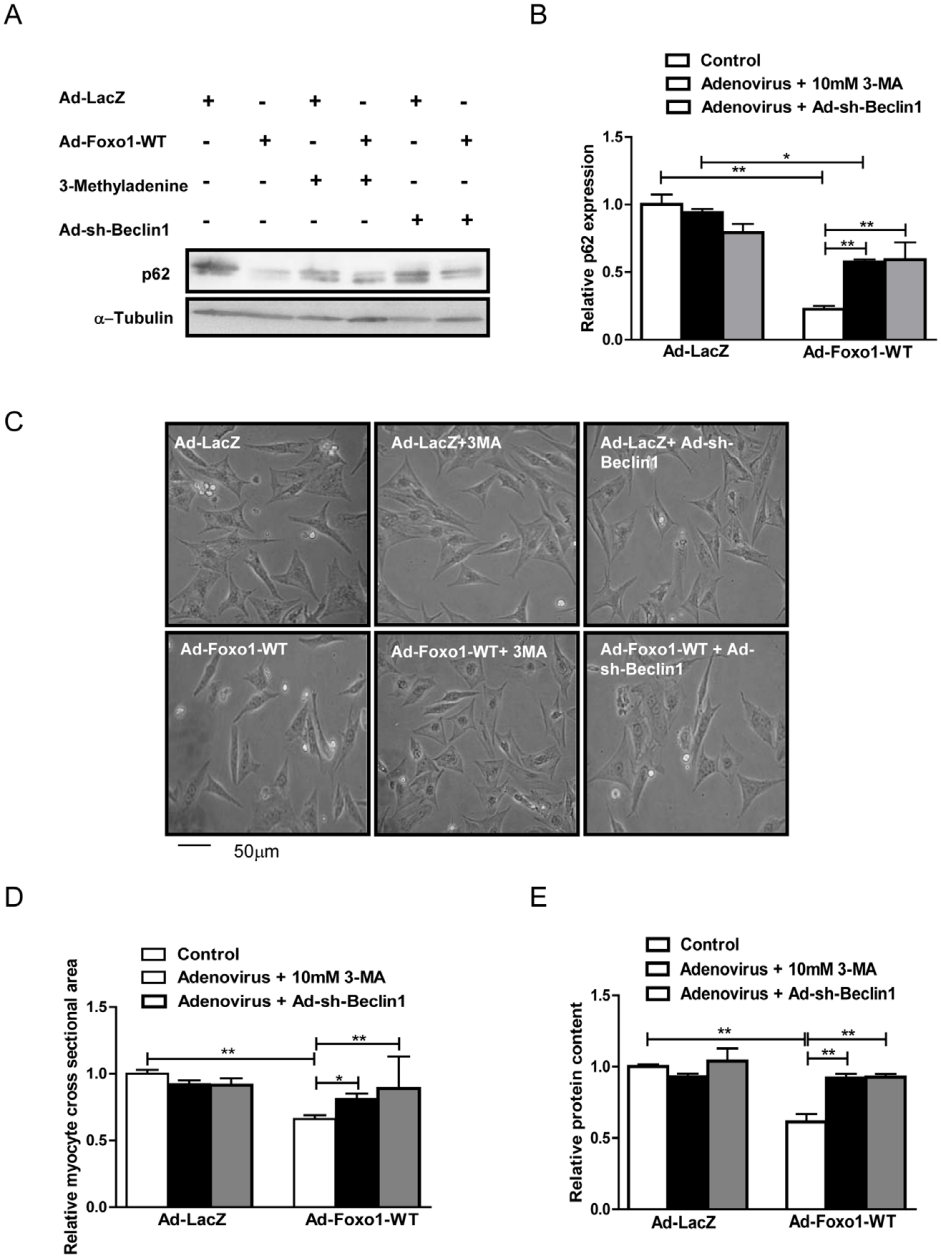
Autophagy inhibition attenuates FoxO1-induced reduction in cell size and relative protein content. Cultured cardiomyocytes were transduced with Ad-FoxO1-WT or Ad-LacZ for 24 hours or Ad-sh-Beclin1 for 96 hours, and treated with 10 mM 3-methyladenine (3-MA) for 24 hours. A) Representative immunoblots. B) Densitometric analyses. C) Representative images of cardiomyocytes viewed under a light microscope. D) Relative cardiomyocyte cross-sectional area. E) Relative protein content. Data represent means from at least 6 different myocyte cultures. * p<0.05, ** p<0.01.

To determine the role of FoxO1 and autophagy in cell size regulation, cultured cardiomyocytes were transduced with an adenovirus harboring WT-FoxO1 (Ad-FoxO1-WT) and treated with known inhibitors of autophagy, 3-methyladenine (3-MA), an inhibitor of class III phosphatidyl inositol -3 kinase (PI3K), or an adenovirus harboring shRNA-Beclin1 (Ad-sh-Beclin1), as described previously [18]. Overexpression of FoxO1 induced autophagy, as seen by p62 degradation (Fig. 4AB), which was significantly attenuated by treatment with 3-MA and Ad-sh-Beclin1 transduction (Fig. 4AB), confirming that 3-MA and Ad-sh-Beclin1 can inhibit FoxO1-induced increases in autophagy. Under these experimental conditions, Ad-FoxO1-WT reduced the cardiomyocyte size (Fig. 4CD) and the relative protein content (Fig. 4E) significantly, while inhibition of autophagy attenuated both of these FoxO1-induced reductions (Fig. 4CDE). Collectively, these results indicate that FoxO1 reduces the size of cardiomyocytes and that autophagy plays an important role in mediating FoxO1-induced decreases in the cell size of cardiomyocytes.

### FoxO1 expression and autophagy are increased in an *in vitro* model of regression of cardiac hypertrophy

**Figure 5:**
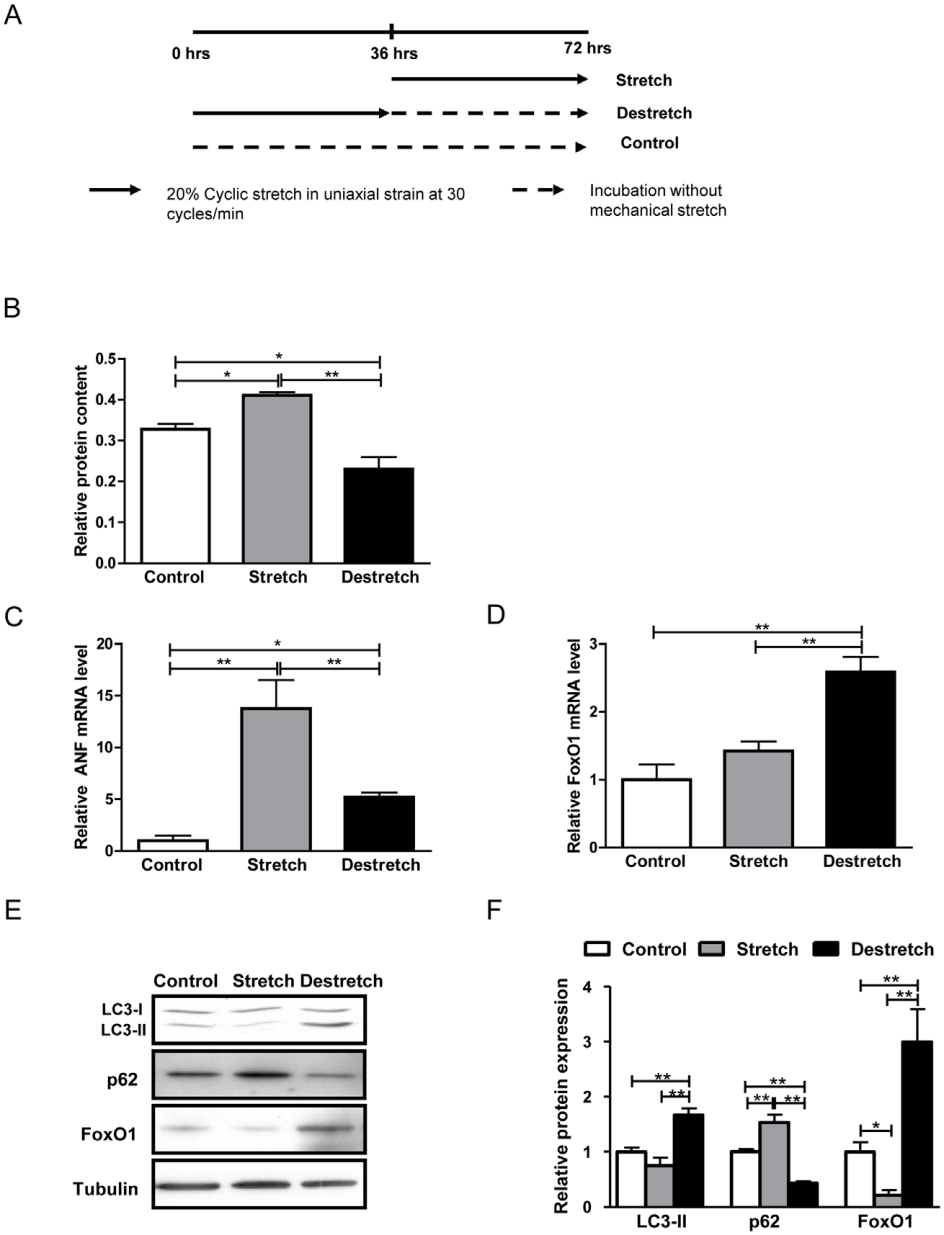
FoxO1 expression and autophagy are increased in an *in vitro* model of regression of cardiac hypertrophy. Cardiomyocytes were cultured in BioFlex plates, subjected to mechanical cyclic stretch for 36 hours (Stretch) and incubated without stretch for 36 hours (de-stretch). A) Scheme showing the regimen of mechanical stretch and de-stretch. B) Relative protein content. C–D) mRNA levels of ANF and FoxO1, determined by qRT-PCR. E) Representative immunoblots. F) Densitometric analyses. Data represent means from at least 4 different myocyte cultures. * p<0.05, ** p<0.01.

To better understand the role of FoxO1 in mediating regression of cardiac hypertrophy, we created an *in vitro* model of regression of cardiac hypertrophy in which cardiomyocytes were cultured in collagen-I-coated special culture dishes, subjected to repetitive mechanical stretch for 36 hours and subsequently incubated without stretch (de-stretch) for the same length of time (Fig. 5A). The relative protein content and the mRNA level of ANF were significantly increased following mechanical stretch, indicating induction of cardiac hypertrophy, but were attenuated after de-stretch, indicating regression of cardiac hypertrophy (Fig. 5BC). FoxO1 mRNA (Fig. 5D) and protein expression levels (Fig. 5EF) were significantly increased after de-stretch. Autophagy was also enhanced during de-stretch, as indicated by increased LC3-II and decreased p62 expression (Fig. 5EF). Taken all together, these results indicate that FoxO1 and autophagy may contribute to regression of cardiac hypertrophy *in vitro.*

### Absence of FoxO1 and autophagy attenuates the extent of regression of cardiac hypertrophy *in vitro* and *in vivo*

**Figure 6:**
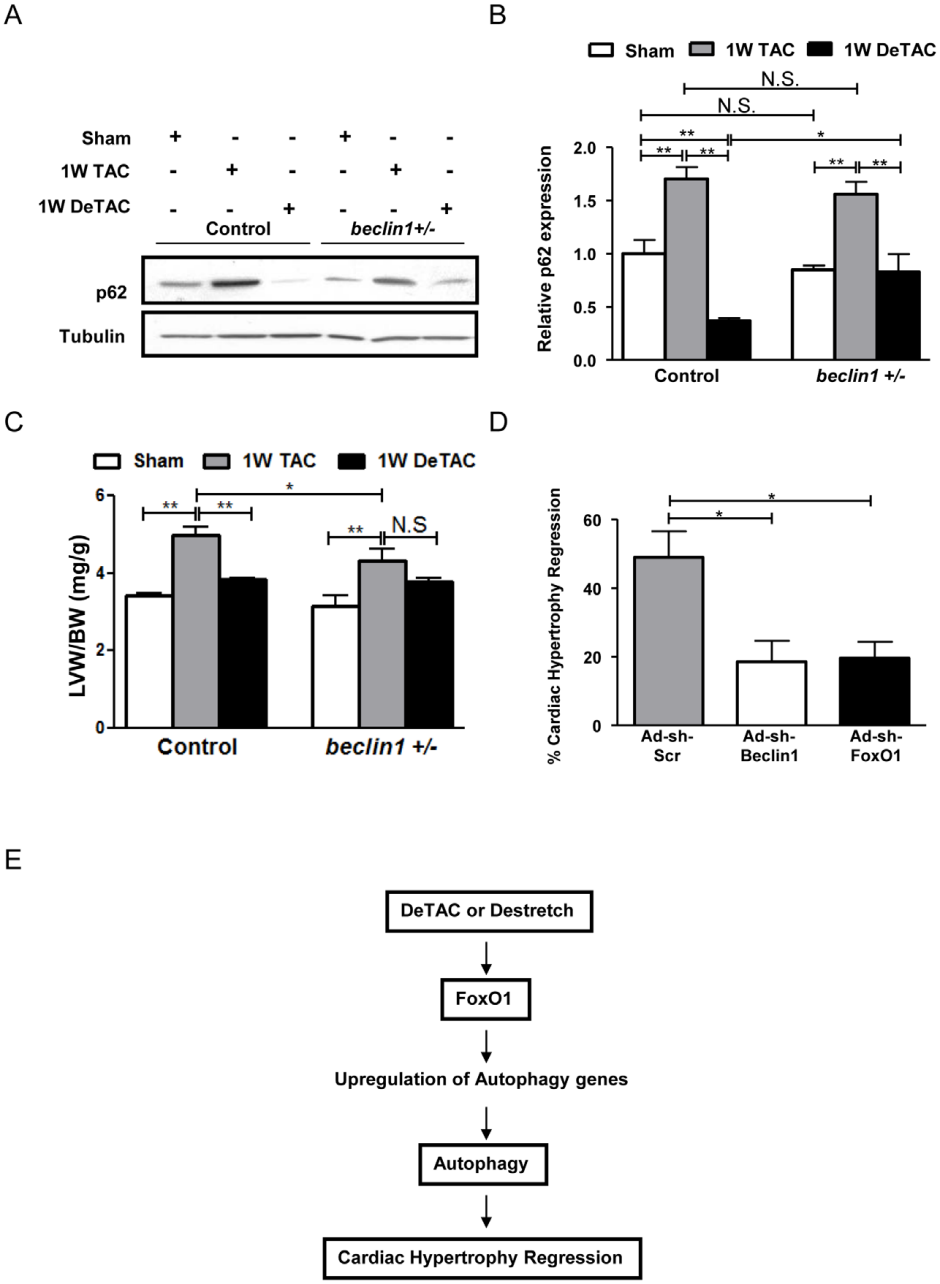
FoxO1 and autophagy are required for regression of cardiac hypertrophy. Control C57BL/6 mice and mice with heterozygous knockout of Beclin1 (*beclin1+/−*) were subjected to 1W TAC and 1W DeTAC surgeries. A) Representative immunoblots. B) Densitometric analyses. C) Left ventricular weight/body weight (LVW/BW). D) Cultured cardiomyocytes were transduced with Ad-sh-Scr, Ad-sh-Beclin1 and Ad-sh-FoxO1 and subjected to mechanical stretch/de-stretch. The percentage reduction in relative protein content after de-stretch compared to after stretch is represented as % regression of cardiac hypertrophy *in vitro*. E) A scheme showing the proposed hypothesis of this study. * p<0.05, ** p<0.01, N.S.: Not Significant.

To show that autophagy is required for mediating regression of cardiac hypertrophy, we subjected *beclin1+/−* mice to regression of cardiac hypertrophy. Autophagy was significantly suppressed in *beclin1+/−* mice after 1W DeTAC, as indicated by increased p62 expression, relative to control mice subjected to 1W DeTAC (Fig. 6AB). In the absence of autophagy, the extent of regression of cardiac hypertrophy was attenuated, as indicated by the significant reduction in LVW/BW after DeTAC in control mice, but not in *beclin1*+/− mice and the decreased % regression of cardiac hypertrophy (which is the percentage decrease in LVW/BW values after 1W DeTAC compared to after 1W TAC alone) (Supplemental Fig. S4) in *beclin1+/−* mice. Similarly, in the absence of autophagy *in vitro* due to knock-down of Beclin1 using Ad-sh-Beclin1, the % regression of cardiac hypertrophy (which is the percentage reduction in relative protein content after de-stretch compared to stretch alone) was significantly attenuated (Fig. 6D). This confirmed that autophagy is required for regression of cardiac hypertrophy. To determine whether FoxO1 is required to mediate regression of cardiac hypertrophy, we used an adenovirus harboring sh-FoxO1 (Ad-sh-FoxO1), which has been described previously [9]. The absence of FoxO1 significantly decreased the extent of regression of cardiac hypertrophy *in vitro* (Fig. 6D). Taken all together, this suggests that endogenous FoxO1 is required for mediating regression of cardiac hypertrophy, possibly through stimulation of autophagy (Fig. 6E).

## Discussion

We here show that a) regression of cardiac hypertrophy is induced by left ventricular unloading and de-stretch of cultured cardiomyocytes after mechanical stretch, and is accompanied by increased autophagy and upregulation of FoxO1, b) FoxO1 increases the expression of autophagy genes and autophagosome formation in mouse hearts *in vivo*, c) overexpression of FoxO1 reduces cardiomyocyte size, whereas inhibition of autophagy attenuates FoxO1-induced reductions in cell size and protein content, and d) autophagy and FoxO1 are required to mediate regression of cardiac hypertrophy both *in vitro* and *in vivo.*

Upregulation of FoxO1 is sufficient to stimulate autophagy in cardiomyocytes *in vitro*, as shown in our previous study [9], and *in vivo*, as shown in this study. We and others have shown previously that FoxO3 also stimulates autophagy in cardiomyocytes [9], [10]. In addition, both FoxO1 and FoxO3 negatively regulate cardiac hypertrophy [14]. Thus, we do not exclude the role of FoxO3, another major isoform of the FoxO family expressed in the heart, in mediating autophagy and consequently mediating regression of cardiac hypertrophy by DeTAC or destretch. However, downregulation of FoxO1 significantly attenuated DeTAC- and de-stretch-induced regression of cardiac hypertrophy, suggesting that FoxO1 may have non-overlapping functions compared to FoxO3 that induce regression of cardiac hypertrophy.

Cardiac hypertrophy induced by pressure overload or mechanical stretch is accompanied by activation of Akt through phosphatidylinositol 3 kinase. Akt phosphorylates FoxOs, thereby inducing their cytosolic translocation, which may remove the FoxOs' negative constraint upon hypertrophy and, thus, induce cardiac hypertrophy [15]. We here demonstrate that protein expression of FoxO1 is upregulated in response to DeTAC or de-stretch and that endogenous FoxO1 is required for regression of cardiac hypertrophy. Whether posttranslational modifications of FoxOs, such as dephosphorylation and deacetylation, are required for activation of autophagy and consequent regression of cardiac hypertrophy remains to be investigated.

FoxO proteins are critical regulators of protein degradation mechanisms, including the autophagy-lysosome pathway and the ubiquitin proteasome system. Although we showed the involvement of the former in mediating regression of cardiac hypertrophy after DeTAC here, at present, FoxO1-mediated upregulation of atrogin-1/MAFbx, a muscle-specific E3 ubiquitin ligase [12], and its involvement in regression of cardiac hypertrophy after DeTAC cannot be excluded. Increasing lines of evidence suggest that functional interactions exist between autophagy and the ubiquitin proteasome system [23]. For example, degradation of ubiquitinated protein by the lysosome may occur through autophagy, with interaction with p62 leading to the ubiquitinated protein being engulfed by autophagosomes.

Overexpression of FoxO1 in the heart markedly reduces cardiac mass and cardiomyocyte size in transgenic mice. It has been shown that FoxO3 negatively regulates cell cycle/proliferation, thereby inhibiting normal growth of the heart during development [24]. We speculate that the small heart observed in our Tg-FoxO1 may be caused by multiple mechanisms activated through persistent upregulation of FoxO1 in postnatal hearts.

The molecular mechanism through which activation of autophagy contributes to regression of cardiac hypertrophy remains to be elucidated. For example, activation of autophagy may allow cardiomyocytes to degrade substantial amounts of proteins and damaged organelles. However, contributions of total proteins degraded by the lysosome to the total level of hypertrophy regression remain to be elucidated. It is formally possible that autophagy may contribute to hypertrophy regression through degradation of signaling molecules or key regulators of protein synthesis/degradation. We show that autophagy may be suppressed by stretch and 1W TAC. However, suppression of autophagy alone may not be sufficient to induce cardiac hypertrophy since some loss-of-function mouse models of autophagy, including *beclin1+/−* mice, do not exhibit cardiac hypertrophy at baseline, at least at a young age. Thus, further research is necessary to clarify the molecular mechanism through which autophagy regulates development and regression of cardiac hypertrophy.

In this model, we were not able to extend the period of the initial TAC sufficiently long enough for the mouse to develop heart failure since the longer TAC facilitated scar formation in the area of constriction and prevented the removal of the pressure gradient. Thus, in order to investigate how regression of cardiac hypertrophy mediated through autophagy affects LV function, improvement of the animal model appears to be essential.

## Supporting Information

Figure S1 Distribution of cardiomyocyte cell size in mice after TAC and DeTAC. C57BL/6 mice were subjected to pressure overload caused by thoracic aortic constriction for 1 week (1W TAC), followed by cardiac unloading by removal of the constriction for 1 week (1W DeTAC). Size distribution of cardiac myocyte cross- sectional area was measured from at least 50 cells from 3 different animals in each group.
(TIF)

Figure S2 Characterization of Tg-FoxO1. Cardiac phenotype of Tg-FoxO1 (line #8). Tg-FoxO1 and non-transgenic (NTg) mice were euthanized at the age of 3 months. A) Pictures of NTg and Tg-FoxO1 (line #8) hearts. Each graduation in the scale below = 1 mm. B) Left ventricular weight (LVW)/body weight (BW) (mg/g). n = 32. C,D) LV cardiomyocyte cross sectional area. Wheat germ agglutinin staining was performed and average cardiomyocyte cross sectional area was obtained in NTg and Tg-FoxO1 hearts.
(TIF)

Figure S3 Stimulation of autophagy in Tg-FoxO1 hearts. Tg-FoxO and NTg mice were treated with chloroquine (Chq, 10 mg/kg, ip), and euthanized 4 hours after treatment. A. Immunoblots showing FoxO1, LC3 and tubulin in the heart. The level of α-tubulin is shown as a loading control. B. Densitometric analyses for LC3-II expression. The data are mean of two experiments.
(TIF)

Figure S4 Regression of cardiac hypertrophy was blunted in *beclin1*+/− mice. Control C57BL/6 mice and transgenic mice with heterozygous knockout of Beclin1 (*beclin1+/−*) were subjected to 1W TAC and 1W DeTAC surgeries. Percentage decrease in LVW/BW values after 1W TAC and 1W DeTAC compared to after 1W TAC alone is represented as % regression of cardiac hypertrophy *in vivo*. **p<0.01.
(TIF)

Table S1 Echocardiographic analyses of mice after TAC and DeTAC.
(TIF)
