# Supplementary figures and images for: Autophagy Plays an Essential Role in Mediating Regression of Hypertrophy during Unloading of the Heart
Source: PLoS One. 2013 Jan 7;8(1):e51632. doi: 10.1371/journal.pone.0051632 (PMC3538681; doi:10.1371/journal.pone.0051632)

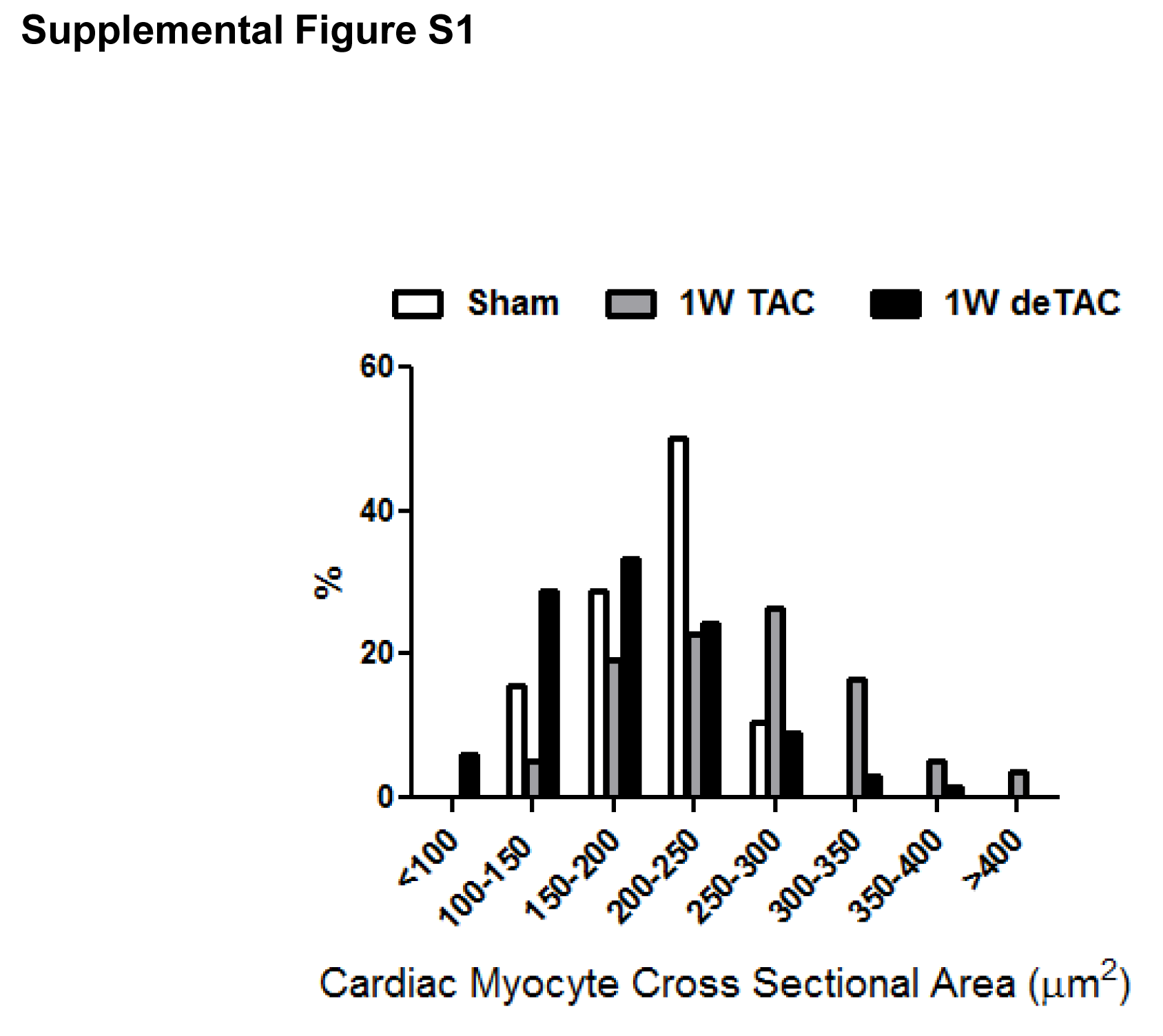

Supplement: Figure S1 — Distribution of cardiomyocyte cell size in mice after TAC and DeTAC. C57BL/6 mice were subjected to pressure overload caused by thoracic aortic constriction for 1 week (1W TAC), followed by cardiac unloading by removal of the constriction for 1 week (1W DeTAC). Size distribution of cardiac myocyte cross- sectional area was measured from at least 50 cells from 3 different animals in each group. (TIF) [file pone.0051632.s001.tif]

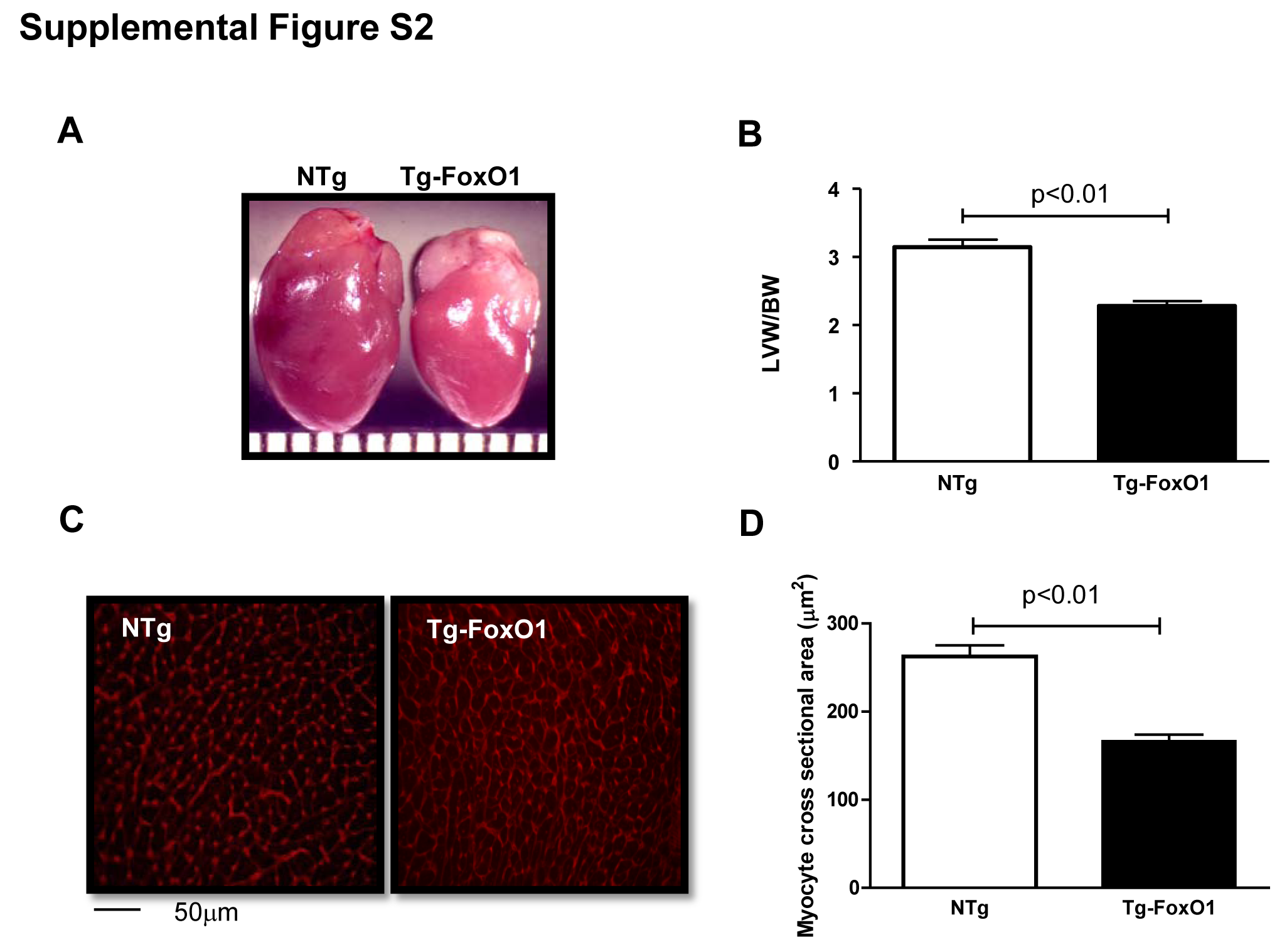

Supplement: Figure S2 — Characterization of Tg-FoxO1. Cardiac phenotype of Tg-FoxO1 (line #8). Tg-FoxO1 and non-transgenic (NTg) mice were euthanized at the age of 3 months. A) Pictures of NTg and Tg-FoxO1 (line #8) hearts. Each graduation in the scale below = 1 mm. B) Left ventricular weight (LVW)/body weight (BW) (mg/g). n = 32. C,D) LV cardiomyocyte cross sectional area. Wheat germ agglutinin staining was performed and average cardiomyocyte cross sectional area was obtained in NTg and Tg-FoxO1 hearts. (TIF) [file pone.0051632.s002.tif]

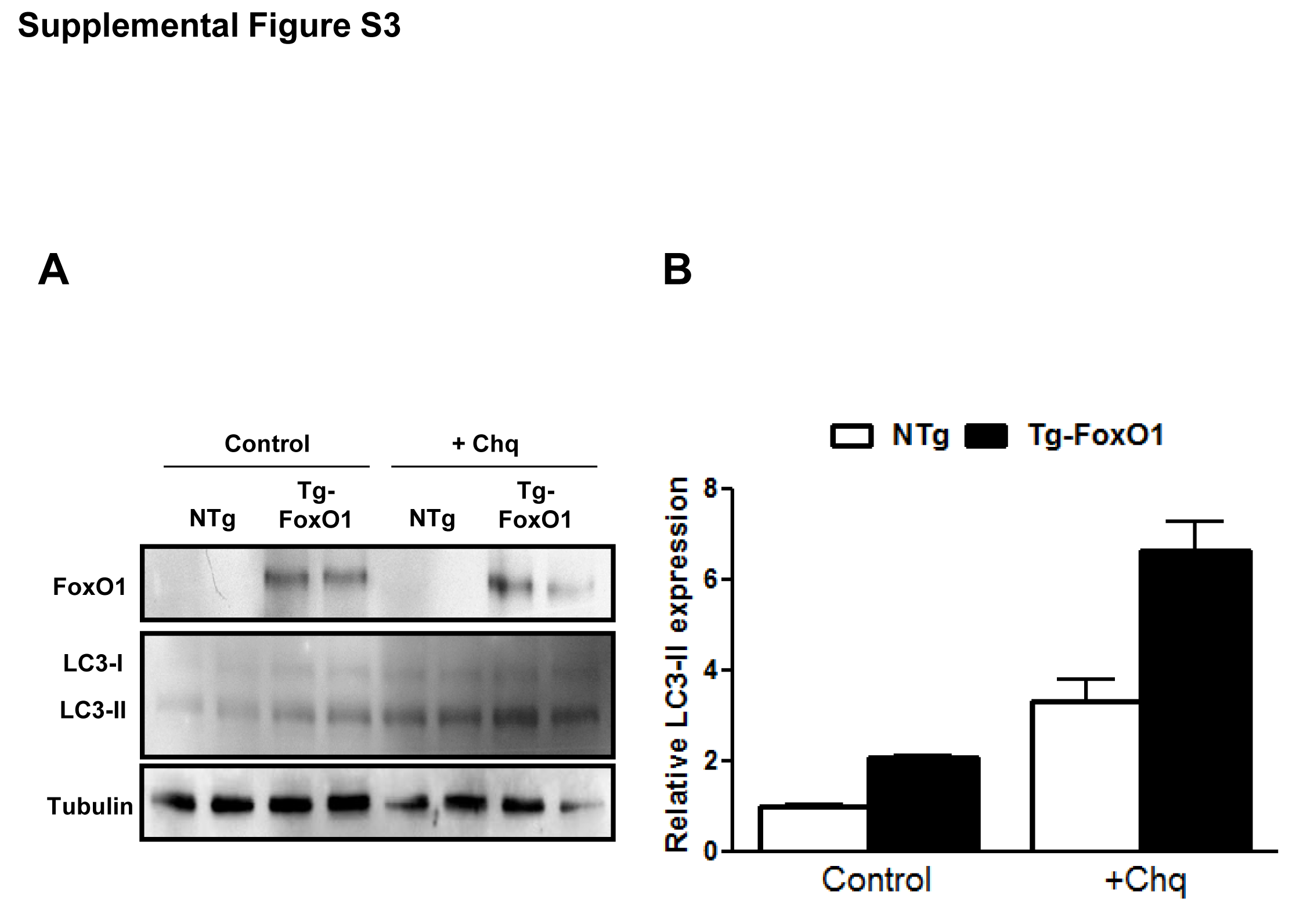

Supplement: Figure S3 — Stimulation of autophagy in Tg-FoxO1 hearts. Tg-FoxO and NTg mice were treated with chloroquine (Chq, 10 mg/kg, ip), and euthanized 4 hours after treatment. A. Immunoblots showing FoxO1, LC3 and tubulin in the heart. The level of α-tubulin is shown as a loading control. B. Densitometric analyses for LC3-II expression. The data are mean of two experiments. (TIF) [file pone.0051632.s003.tif]

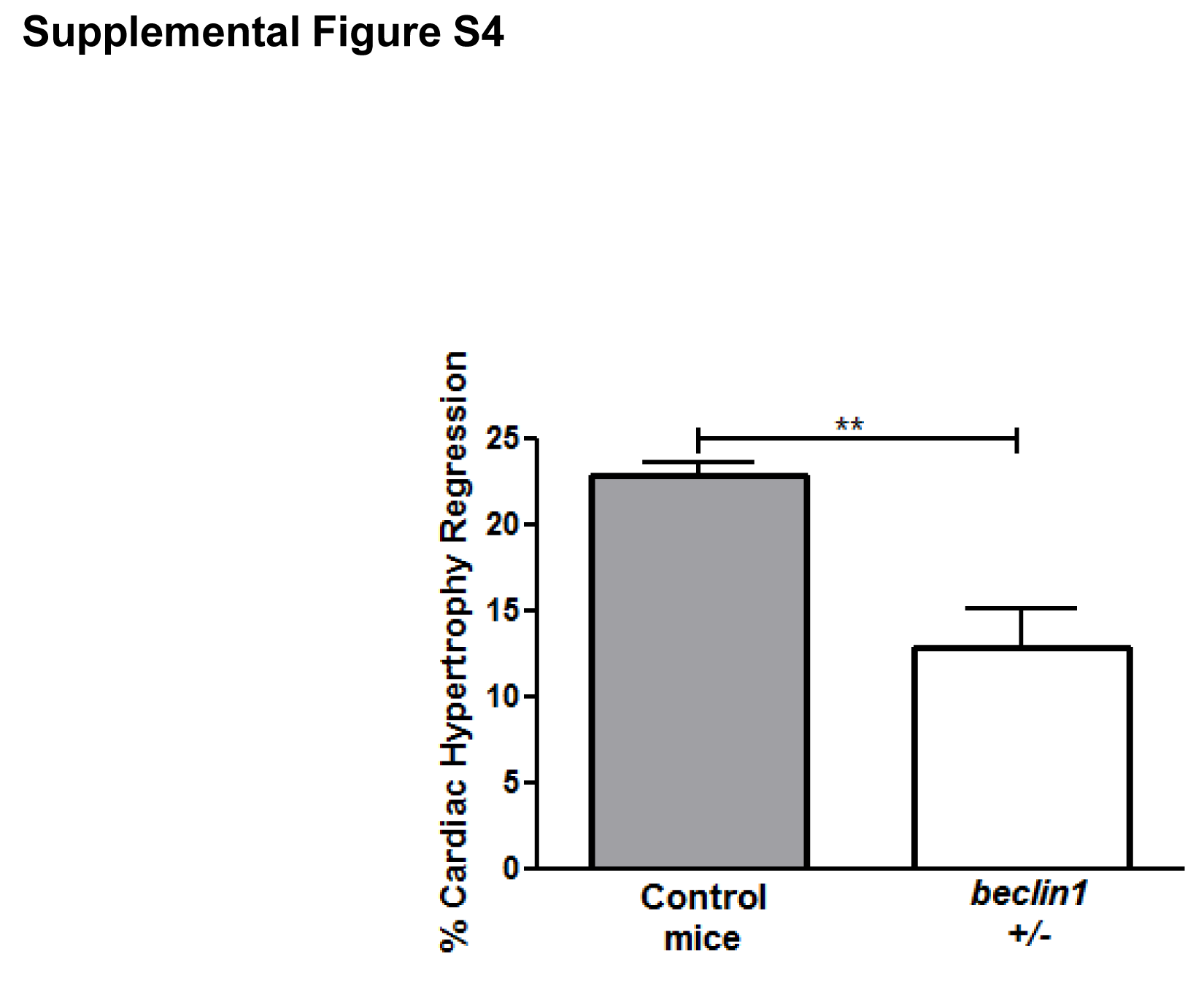

Supplement: Figure S4 — Regression of cardiac hypertrophy was blunted in beclin1+/− mice. Control C57BL/6 mice and transgenic mice with heterozygous knockout of Beclin1 (beclin1+/−) were subjected to 1W TAC and 1W DeTAC surgeries. Percentage decrease in LVW/BW values after 1W TAC and 1W DeTAC compared to after 1W TAC alone is represented as % regression of cardiac hypertrophy in vivo. **p<0.01. (TIF) [file pone.0051632.s004.tif]

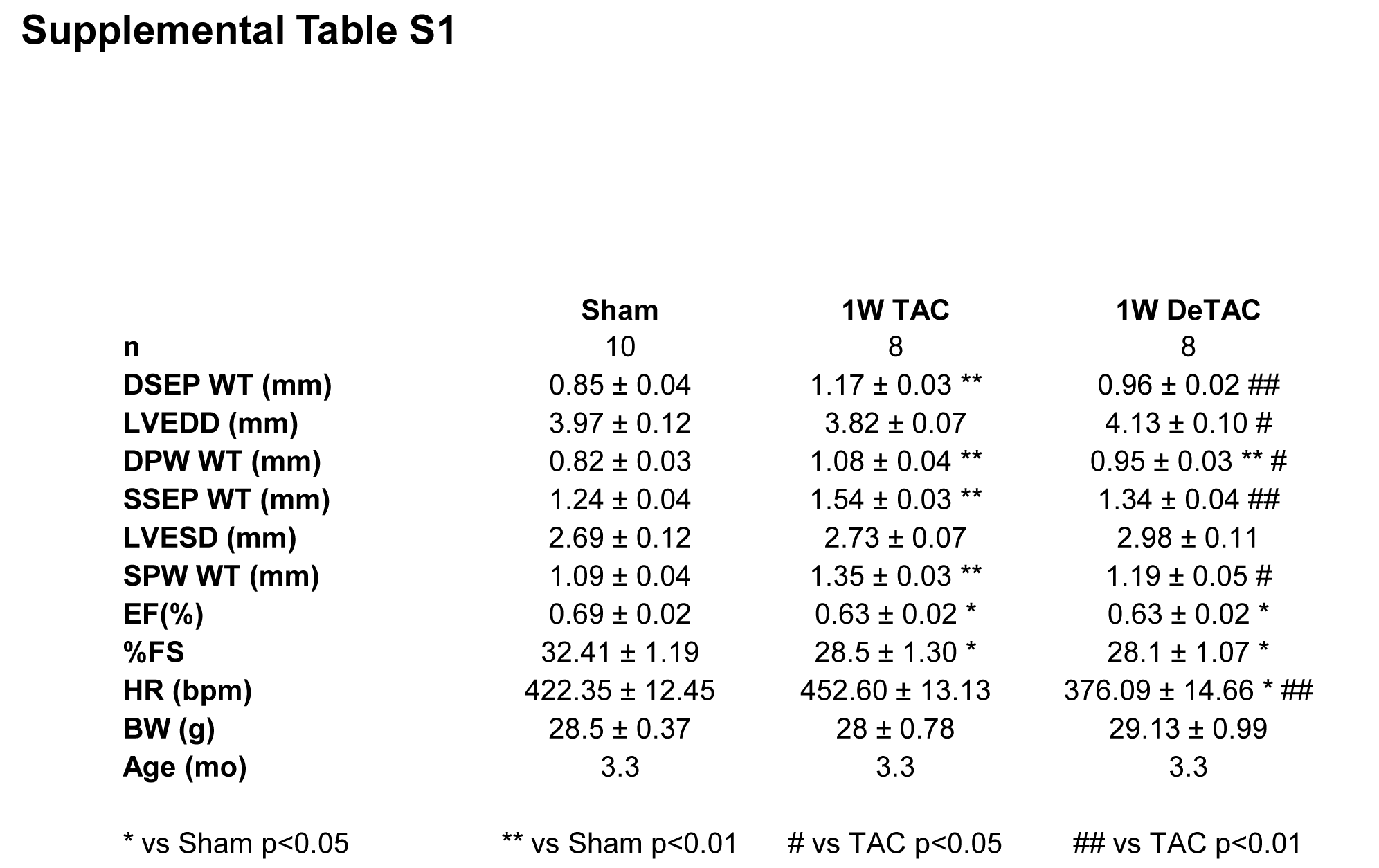

Supplement: Table S1 — Echocardiographic analyses of mice after TAC and DeTAC. (TIF) [file pone.0051632.s005.tif]
